# Morphological Characterization of Human Lung Cancer Organoids Cultured in Type I Collagen Hydrogels: A Histological Approach

**DOI:** 10.3390/ijms241210131

**Published:** 2023-06-14

**Authors:** Irene Monleón-Guinot, Lara Milian, Patricia Martínez-Vallejo, María Sancho-Tello, Mauro Llop-Miguel, José Marcelo Galbis, Antonio Cremades, Carmen Carda, Manuel Mata

**Affiliations:** 1 Department of Pathology, Faculty of Medicine and Dentistry, Universitat de València, 46010 Valencia, Spain; irene.monleon@gmail.com (I.M.-G.); lara.milian@uv.es (L.M.); maria.sancho-tello@uv.es (M.S.-T.); mauro.llop@uv.es (M.L.-M.); antonio.cremades@uv.es (A.C.); carmen.carda@uv.es (C.C.); 2 INCLIVA Biomedical Research Institute, 46010 Valencia, Spain; 3 Hospital de la Ribera, 46600 Alzira, Spain; josegalbiscar@icloud.com; 4 Biomedical Research Networking Center on Bioengineering, Biomaterials and Nanomedicina (CIBER-BBN), 28029 Madrid, Spain; 5 Biomedical Research Networking Center of Respiratory Diseases (CIBERES), 28029 Madrid, Spain

**Keywords:** lung cancer, EMT, CAFS, TME, organoids, in vitro models, 3D culture

## Abstract

The malignity of lung cancer is conditioned by the tumor microenvironment (TME), in which cancer-associated fibroblasts (CAFs) are relevant. In this work, we generated organoids by combining A549 cells with CAFs and normal fibroblasts (NF) isolated from adenocarcinoma tumors. We optimized the conditions for their manufacture in a short time. We evaluated the morphology of organoids using confocal microscopy analysis of F-actin, vimentin and pankeratin. We determined the ultrastructure of the cells in the organoids via transmission electron microscopy and the expression of CDH1, CDH2 and VIM via RT-PCR. The addition of stromal cells induces the self-organization of the organoids, which acquired a bowl morphology, as well as their growth and the generation of cell processes. They also influenced the expression of genes related to epithelial mesenchymal transition (EMT). CAFs potentiated these changes. All cells acquired a characteristic secretory phenotype, with cohesive cells appearing inside the organoids. In the periphery, many cells acquired a migratory phenotype, especially in organoids that incorporated CAFs. The deposit of abundant extracellular matrix could also be observed. The results presented here reinforce the role of CAFs in the progression of lung tumors and could lay the foundation for a useful in vitro pharmacological model.

## 1. Introduction

Lung cancer is the leading cause of cancer mortality worldwide [1]. Among the different types of this cancer, the most common subtype, accounting for 85% of all cases, is non-small cell lung cancer (NSCLC), including adenocarcinoma (ADC), which accounts for 40% of all cases [2]. To reduce mortality from lung cancer, early detection and prevention of lung cancer are crucial, as well as the development of new therapies based on a better understanding of lung carcinogenesis [3].

The high mortality of NSCLC is related to the acquisition of resistance to chemotherapy, as well as the great invasiveness and metastatic capacity of these tumor cells. On the other hand, the pathogenesis of NSCLC is determined by the interactions of the different elements that constitute the so-called tumor microenvironment (TME) [4,5], which comprises the extracellular matrix (ECM) and stromal cells, including cancer-associated fibroblasts (CAFs) among others [6]. The interstitial ECM is aberrantly enriched and remodeled in the tumor microenvironment and promotes tumor invasion [7,8]. The main component of the ECM in lung cancer is collagen, mainly type I collagen (Col-1) [9], which is secreted by fibroblasts and may also be secreted by cancer cells, and is important for tumor growth and epithelial-mesenchymal transition (EMT) [9].

The ability to predict the evolution or response to treatment in lung cancer is very limited, partly due to the lack of good models to study the different elements that make up the TME. On the one hand, traditional 2D cultures of standard cell lines that have been widely used have proven ineffective in clinical studies due to the inability of such cultures to faithfully recapitulate the complexity of patients’ tumors [10,11,12]. On the other hand, cancer cell lines generally do not maintain the genetic heterogeneity of the original tumors [11,13].

Three-dimensional (3D) organoids are a better representation of in vivo physiological conditions than 2D cultures, while retaining many of the advantages of cell culture [11]. Organoids are 3D structures composed of multiple cells of the primary tissue, specific to the organ of origin, which they resemble in terms of structure and function in vivo [14,15]. With respect to lung cancer, organoids have become a promising model for the study of interactions between the different elements of the TME.

Organoids can be cultured directly from tumors or constructed according to known cellular or matrix elements. For some authors, organoid culture represents a better option for the study of lung cancer since it better reflects the epigenetic or histological peculiarities of this pathology. However, the possibility of generating organoids by choosing a certain composition should allow us to better study the role of each element that makes up the TME [16,17].

The terms organoid, spheroid and 3D cell culture have been used interchangeably in the literature. Spheroids are spherical units of cells that are generally cultured as free-floating aggregates and are arguably of low complexity in mirroring tumor organization. In general, organoids can be referred to as cells that grow in 3D to form structural units that partially resemble the organ, both in structure and function [18]. The use of in vitro study systems based on organoids or spheroids has made it possible to establish the role of stromal cells in the control of the assembly, migration, or metastasis of tumor cells [19]. With this background, the design of 3D culture systems that incorporate stromal cells as CAFs could be useful to establish pharmacological trial systems with the philosophy of personalized medicine. To the best of our knowledge, this fact is not sufficiently explored in the NSCLC.

Different researchers have used cultures of spheroids or organoids to study the expression of certain markers, extracellular matrix proteins, EMT, etc. However, there is no research focused on the standardization of morphological parameters in the development of NSCLC organoids, which is one of the objectives of our research.

Thus, the aim of the present study is to standardize the culture conditions as well as the morphological parameters related to the growth, distribution, and invasion of organoids generated from stromal cells isolated from lung adenocarcinomas and from A549 cells, to generate a platform for in vitro pharmacological trials that allows the prediction of the effect of different drugs on parameters related to tumor growth and invasion. These effects could potentially be tested in individual patients in the context of personalized medicine.

## 2. Results

### 2.1. Optimization of Spheroid/Organoid Manufacture

**Figure 1:**
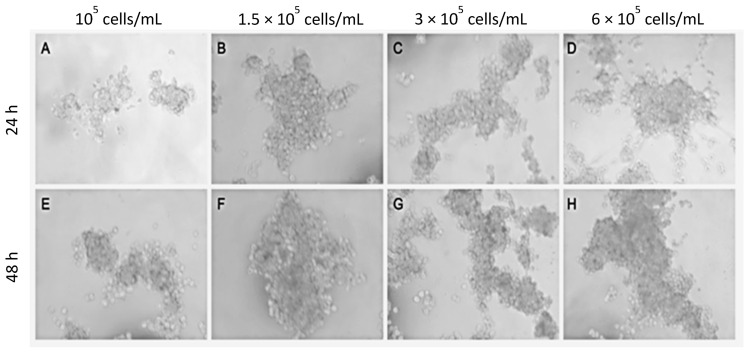
Optimization of the initial cell concentration. The cell suspension containing a mixture of A549 cells and CAFs (50/50) were diluted to different concentrations and cultured for 24 h (**A**–**D**) or 48 h (**E**–**H**) using the hanging drop method. Representative phase contrast images from *n* = 3 separate experiments are shown.

Our first objective was to optimize the experimental conditions regarding the optimal cell density and culture time to be used in the hanging drop method, using A549 cells and CAFs. Both cell types were mixed (50% each) at densities between 10^5^ and 6 × 10^5^ cells/mL in 50 µL hanging drops, cultured for 48 h, and their morphology was inspected via phase contrast microscope every 24 h. Representative results are shown in Figure 1. Organoids constructed with cell densities below 1.5 × 10^5^ cells/mL were small and non-homogeneous (Figure 1A,E). In those of 1.5 × 10^5^ cells/mL, the cells formed groups of a homogeneous size of 60–80 µm in diameter (Figure 1B,F). At higher cell density, cell groups with heterogeneous size diameter were observed, some of which were very small (around 40 µm), while others were very large (greater than 120 µm) (Figure 1C,D,G,H). In view of these results, we decided to use the cell density of 1.5 × 10^5^ cells/mL for the rest of the experiments.

**Figure 2:**
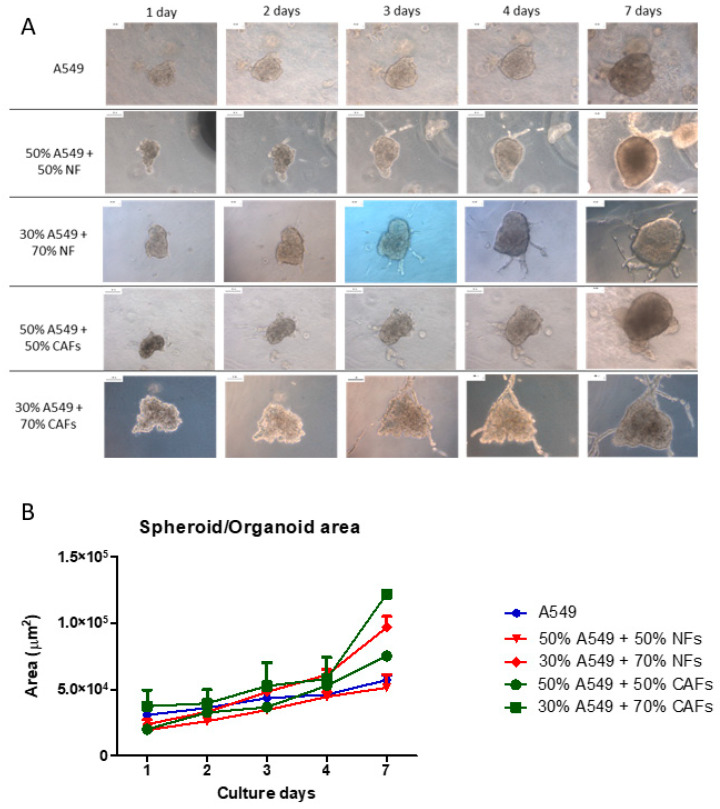
Evolution of the size of organoids/spheroids in type I collagen hydrogels. (**A**) Spheroids of A549 cells, NFs or CAFs, as well as organoids of A549 cells + NFs or A549 cells + CAFs were constructed and cultured in type I collagen hydrogels for 1 week. Representative phase contrast images of *n* = 3 separate experiments are shown. Scale bar = 100 µm. (**B**) The mean area ± SD of *n* = 3 different organoids is shown.

Spheroids from A549 cells, CAFs or NFs, as well as organoids from A549 cells + CAFs or A549 cells + NFs were manufactured. They were collected with a Pasteur pipette and resuspended in 50 µL drops of type I collagen hydrogel, which were incubated at 37 °C after complete reticulation and then covered with culture medium. The constructs were cultured for 1 week and images of representative organoids were acquired daily, always photographing the same organoid at the same magnification to minimize variability. Experiments were performed in triplicate and five different organoids were selected from each experimental condition. Representative results are shown in Figure 2. The areas of at least 5 different spheroid/organoids were calculated (Figure 2B). The A549 cells formed well-defined spheroids that included epithelial cells of characteristic appearance, which maintained a clear cohesion between them. These spheroids grew appreciably and showed cells with an epithelioid morphology that migrated towards the periphery of the hydrogel. NFs and CAFs generally did not form spheroids but expanded through the collagen scaffold. NFs only formed spheroids in exceptional cases, but they did not show appreciable growth. CAFs formed spheroids more frequently than NFs, although this frequency was also very low. CAFs spheroids showed a large size and appreciable growth in culture. In addition, these tumor fibroblast cells showed marked invasion of the scaffold.

Organoids with two different proportions of A549 cells and NFs were generated. Organoids generated with 50% of each cell type did not experience a significant increase in area, compared to spheroids from A549 cells (Figure 2). However, organoids generated with an increase in the NFs ratio to 70% showed considerable growth relative to A549 cells spheroids. Moreover, cellular processes could be observed in these organoids, as well as an invasion of the scaffolds by both epithelioid and fibroblastic cells.

In the case of the organoids generated with CAFs, a significant increase in their area was observed in all cases with respect to the A549 cell spheroids, being very marked when 70% CAFs were used. In the same way as with NFs, cellular processes could be observed arising from the organoid, which in this case were longer and more branched than those observed with NFs organoids. Likewise, a notable number of migratory cells with different morphologies could be observed (Figure 2).

### 2.2. Spheroid Morphology of A549 Cells

**Figure 3:**
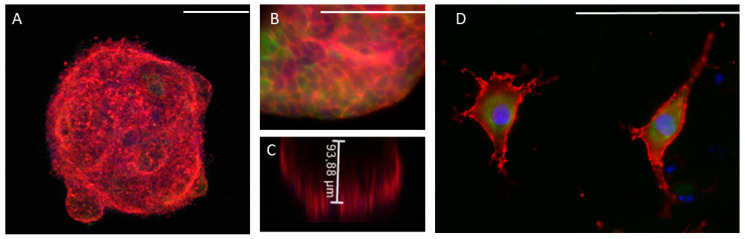
Morphology of A549 cells spheroids. Spheroids were constructed and cultured in type I collagen scaffolds for one week. F-actin distribution was analyzed via phalloidin-rhodamine fluorescence staining (red). Pankeratin expression was detected by immunofluorescence (green). DAPI stains nuclei blue. Samples were studied under confocal (**A**,**C**) or epifluorescence (**B**,**D**) microscopy. The height of the organoids was estimated by confocal microscopy in the Z axis (**C**). Results are representative of *n* = 3. Scale bar = 100 µm.

A549 cells formed spheroids that in all cases maintained their cohesion when cultured in type I collagen scaffolds. The spheroids grew significantly throughout the week of culture, acquiring a compact morphology, with well-defined limits and few cells migrating outside of it (Figure 3A, Supplementary Material Video S1). These spheroids generally reached a height close to 100 µm (Figure 3C). Regarding the morphology of the cells that make up the spheroids, those cells inside the spheroids showed a characteristic epithelioid morphology, with good intercellular cohesion and characteristic expression of pankeratin (Figure 3B). However, on the surface of the organoid, migratory cells could be observed that had developed characteristic cell processes, acquiring a fusiform morphology. In addition, we were also able to observe both fibroblastic cells and others with a transitory morphology between epithelial and mesenchymal phenotypes, which showed positivity for vimentin (Figure 3D).

### 2.3. Morphology of NFs and CAFs Spheroids

**Figure 4:**
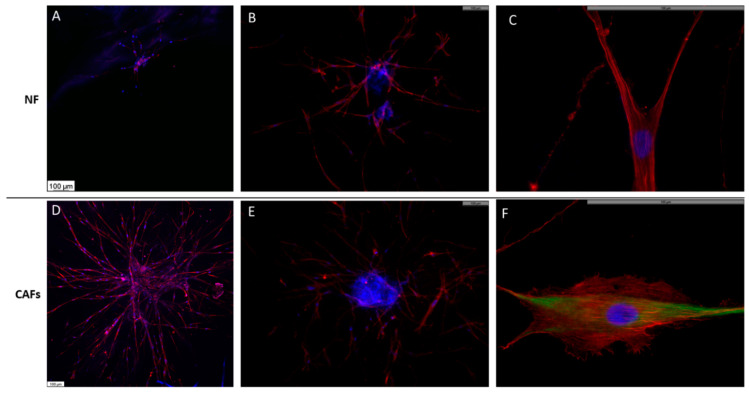
Morphology of NFs (**A**–**C**) or CAFs (**D**–**F**) spheroids. Spheroids were constructed and cultured in type I collagen scaffolds for one week. F-actin distribution was analyzed via fluorescence staining with phalloidin-rhodamine (red). Vimentin expression was studied by immunofluorescence (green). DAPI stains nuclei blue. Samples were studied under an epifluorescence microscope. The results are representative of *n* = 3. Scale bar = 100 µm.

Neither NFs nor CAFs generated cohesive spheroids when cultured in type I collagen hydrogels. Cells tended to migrate rapidly, occupying the entire scaffold, and only exceptionally could some cell aggregation be observed (Figure 4). The cells did not show any cohesion among themselves and acquired a characteristic fibroblastic morphology. The cell clusters made up of NFs contained significantly fewer cells than those made up of CAFs. In the case of the NFs, the cells had a somewhat more spindle-shaped and elongated morphology. The actin acquired a characteristic pattern with thick parallel stress fibers and were oriented in relation to the cell and occupying the central axis of the cell processes, which is the typical pattern of migratory cells (Figure 4B,C). In the case of the CAFs, the cells showed a slightly more developed cytoplasm with abundant vimentin filaments (Figure 4F).

### 2.4. A549 + NFs Organoids Morphology

**Figure 5:**
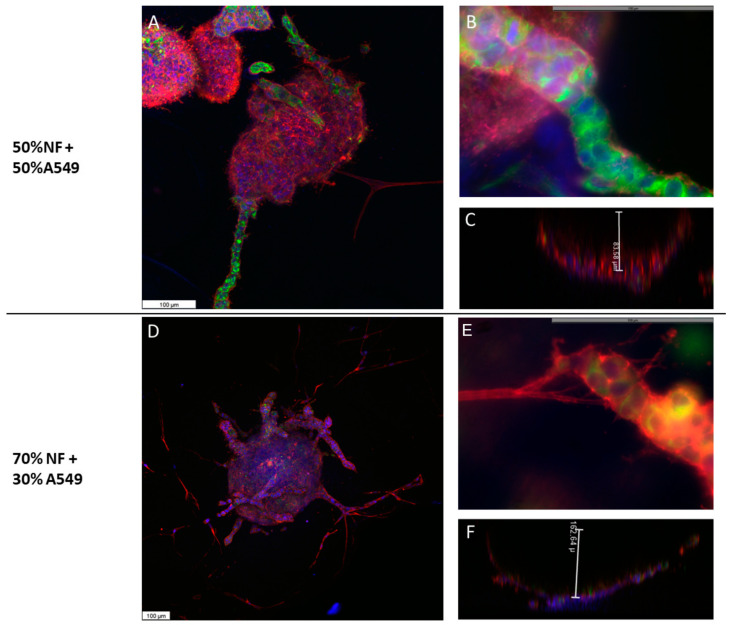
Morphology of A549 cells and NFs organoids. Organoids were made by mixing A549 cells with a 50% (**A**–**C**) or 70% (**D**–**F**) NFs and cultured in type I collagen scaffolds for one week. F-actin distribution was analyzed via phalloidin-rhodamine fluorescence staining (red). Green tracer was used to specifically stain the keratin of A549 cells (green fluorescence). DAPI stains nuclei blue. Samples were studied under confocal microscopy. The height of the organoids was estimated by confocal microscopy in the Z axis (**C**,**F**). Results are representative of *n* = 3. Scale bar = 100 µm.

The morphology of the organoids composed of A549 cells and FNs was radically different from that previously observed in spheroids. The 3D reconstruction of the generated organoids showed that the cells were organized in bowl-shaped alveolar structure, in such a way that the center of the organoid was devoid of cells, while the cells grew from the edges towards the deep part of the collagen droplet (Supplementary Material Video S2). These cells showed typical epithelial morphology, with a regular polygonal shape and a cohesive organization (Figure 5A,D). The average height of these formations was 80 µm (Figure 5C). On some occasions, the organoids reached the surface of the droplet and the cells then spread over the surface of the droplet. The organoids generated with 50% of each cell type occasionally showed cellular processes that expanded in an organized manner to the depth of the scaffold (Figure 5A). Differential immunolabeling showed that the core of these branches consisted almost exclusively of A549 epithelial cells (Figure 5B). As the proportion of NFs used to construct the organoids increased, we observed that, on the one hand, the organoids became larger and deeper (Figure 5D,F), and on the other hand, they presented a greater number of processes that were more branched and that, in addition to containing A549 cells forming the nucleus of the branches, elongated fibroblasts appeared on their periphery and marked the leading edge of such processes (Figure 5E, Supplementary Material Video S3).

### 2.5. A549 + CAFs Organoids Morphology

**Figure 6:**
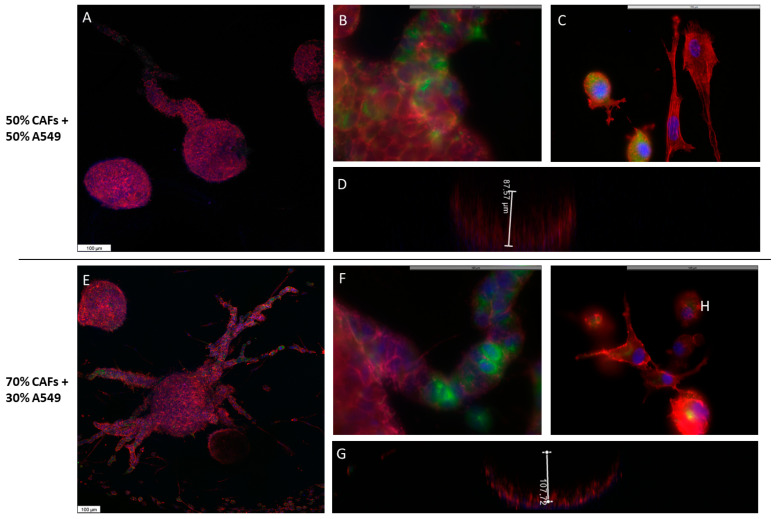
Morphology of A549 cells and CAFs organoids. Organoids were made by mixing A549 cells with 50% (**A**–**C**) or 70% (**D**,**E**) CAFs and cultured in type I collagen scaffolds for one week. F-actin distribution was analyzed via phalloidin-rhodamine fluorescence staining (red). Green tracer was used to specifically stain the keratin of A549 cells (green fluorescence, (**B**,**F**)). Pankeratin and vimentin expression was assayed by immunofluorescence staining ((**C**,**H**), respectively, green fluorescence). DAPI stains nuclei blue. Samples were studied under confocal microscopy. The depth of the organoids was estimated by confocal microscopy in the Z axis (**D**,**G**). The results are representative of *n* = 3. Scale bar = 100 µm.

The morphology of the organoids generated with A549 cells and CAFs was similar to that described for A549 cells and NFs. A much higher number of cellular processes was observed, which increased significantly when the rate of CAFs was increased from 50 to 70% (Figure 6, Supplementary Material Videos S4 and S5). Likewise, many cells were observed around the organoid that eventually generated other smaller organoids. Pankeratin staining showed that some of these cells were epithelial cells at different stages of development of the EMT process, some cells of epithelioid appearance with process development and others of fibroblastic morphology. Along with these cells, pankeratin-negative fibroblast-like cells were observed. All cells at the periphery of the organoids were positive for pankeratin.

### 2.6. Ultrastructural Features of Cells Cultured in Spheroid/Organoids

**Figure 7:**
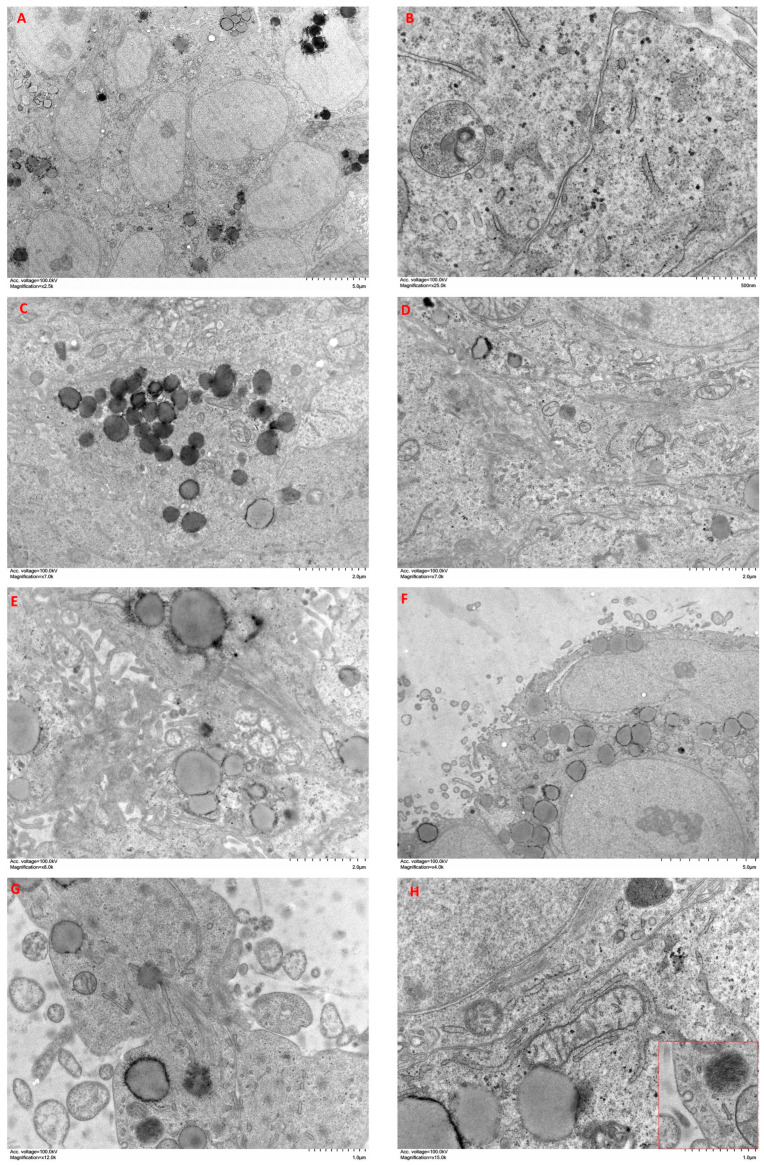
Ultrastructure features of A549 cell spheroids. Spheroids were constructed and cultured in type I collagen scaffolds for one week. Samples were analyzed with TEM. Results shown are representative of *n* = 3 separate experiments.

A549 cells cultured in spheroids showed cohesive clusters of cells with a secretory phenotype (Figure 7A). They presented abundant desmosomes, as well as a well-developed Golgi apparatus and RER (Figure 7B). Many of these cells contained abundant secretory granules of different morphology (Figure 7C). Discrete bundles of actin microfilaments were found inside epithelioid cells (Figure 7D). Within the spheroid, some extracellular spaces were observed surrounding several small cell processes with abundant large actin bundles (Figure 7E). The periphery of the spheroids was occupied by cells with abundant processes and discrete deposits of collagen fibers (Figure 7F). Cellular processes contained abundant bundles of actin microfilaments, as well as some secretory vesicles (Figure 7G). Some cells acquired a fibroblastic spindle morphology, with a decreased secretory phenotype and increased development of the cytoskeleton, as well as caveolae on the surface (Figure 7H inset).

**Figure 8:**
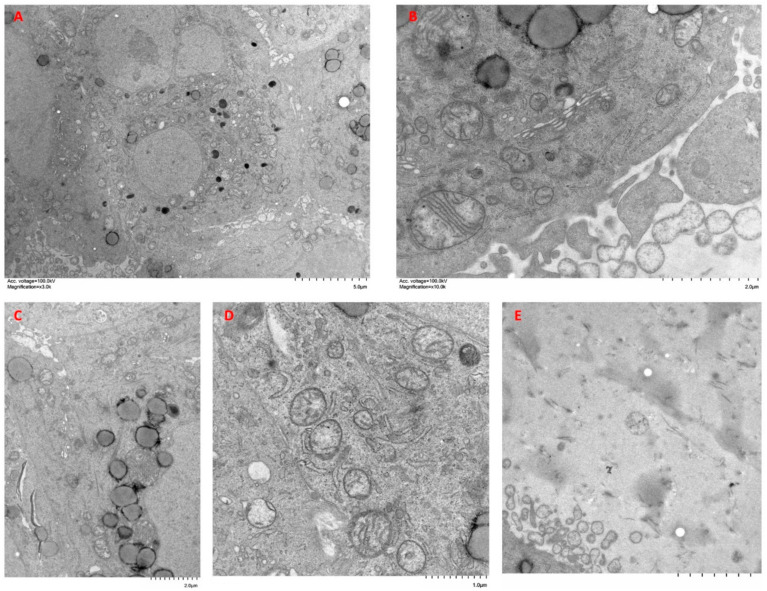
Ultrastructure features of A549 cells + CAFs organoids. Organoids were constructed with A549 cells + CAFs (50/50) and cultured in type I collagen scaffolds for one week. Samples were analyzed with TEM. Results shown are representative of *n* = 3 separate experiments.

Organoids of A549 + CAFs showed heterogeneous clusters of cells with different morphologies (Figure 8A). On the one hand, we highlight cells with epithelioid morphology that contained abundant secretory granules and developed Golgi apparatus and RER (Figure 8B). These cells presented greater accumulation of actin bundles than those of A549 cell spheroids (Figure 8C). On the other hand, we distinguished elongated cells with a lower content of secretory granules and abundant actin bundles (Figure 8D). The periphery of the organoid showed abundant cellular processes, which covered a large area around the organoids, and abundant collagen fiber bundles were observed in the matrix surrounding the organoids (Figure 8E).

**Figure 9:**
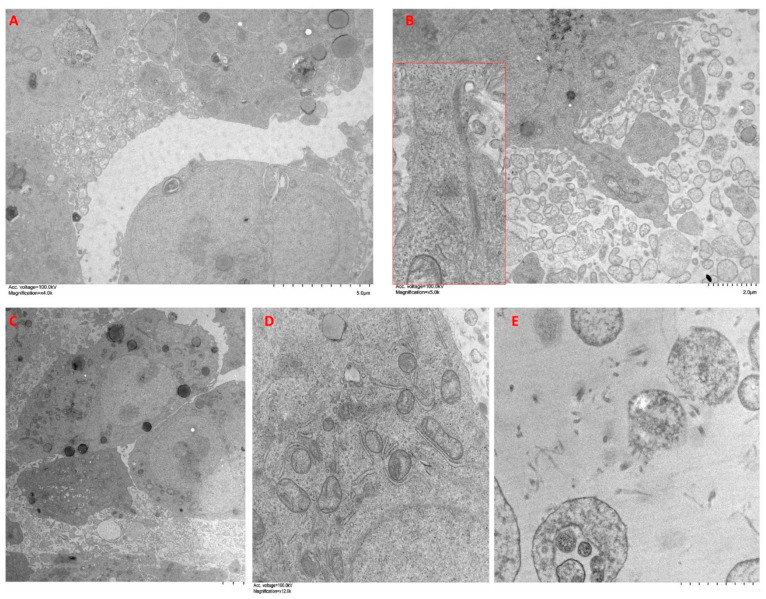
Ultrastructure features of A549 cells + NFs organoids. Organoids were constructed with A549 cells + NFs and cultured in type I collagen scaffolds for one week. Samples were analyzed with TEM. Results shown are representative of *n* = 3 separate experiments.

Organoids of A549 + NFs showed less cohesive clusters of cells and abundant spaces between them, where numerous cell processes were observed (Figure 9A). Both single cells and small groups of cells appeared in the proximity of the organoids (Figure 9B). Some of these cells displayed a characteristic secretory phenotype with abundant RER, Golgi apparatus, mitochondria, and secretory granules (Figure 9C,D), whereas other cells presented a more elongated shape with fewer secretory granules (Figure 9C). At the edge of the organoid, cells emitted long processes into the surrounding matrix. In these processes, a highly developed cytoskeleton was observed along with RER and abundant glycogen (Figure 9B). The matrix surrounding the organoid showed abundant collagen fibers and cellular processes (Figure 9E).

### 2.7. Analysis of the Relative Gene Expression of the Epithelial-Mesenchymal Transition (EMT)

**Figure 10:**
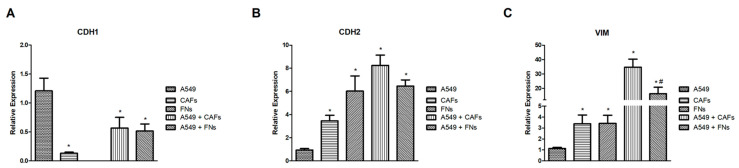
Relative gene expression levels of *CDH1* (**A**), *VIM* (**B**) and *CDH2* (**C**). Spheroids/organoids were generated and cultured in type I collagen hydrogels for 72 h. Relative gene expression was calculated using real-time RT-PCR. Semi-comparative ∆∆Ct method was used to calculated relative changes. A549 cells spheroids was chosen as control group. The *GAPDH* gene was used as housekeeping gene. The mean ± SD of three independent experiments is represented. * *p* < 0.05 versus the control group. # *p* < 0.05 versus A549 cells + CAFs group.

Finally, we wanted to analyze the relative gene expression changes of three well-known EMT markers, namely *CDH1*, *VIM* and *CDH2*. To do this, we generated spheroids of A549 cells, CAFs or FNs, as well as organoids of A549 cells + CAFs or A549 cells + NFs, cultured them in type I collagen scaffolds for 72 h and analyzed the relative gene expression of *CDH1*, *VIM* and *CDH2* via real-time RT-PCR, using the experimental group of A549 cell spheroids as control. As house-keeping gene, we chose *GAPDH*. The results obtained are represented in Figure 10.

Regarding CDH1 (Figure 10A), we did not detect expression in the group of NFs spheroids. In the group of CAFs spheroids, we found low but significant levels of the cDNA codifying for this marker. The co-culture of A549 cells with CAFs or with NFs, showed relative expression levels of *CDH1* significantly lower than the control group, but higher than those observed in the cultures of CAFs or FNs spheroids.

*VIM* gene expression (Figure 10C) was significantly higher in spheroids generated from CAFs or NFs than in those generated with A549 cells. In both organoids assayed, *VIM* gene expression upregulation was significantly higher than in the spheroid groups, reaching maximum values in the spheroids generated with A549 cells and CAFs. A similar trend was found in the case of the *CDH2* gene (Figure 10B), although in this case the differences between the different organoids were not significant.

## 3. Discussion

Lung cancer is one of the leading causes of mortality in developed countries and is the most lethal type of cancer. This is due to multiple factors that involve both the tumor cells themselves and their ability to transition to migratory cells in a process called EMT, as well as the ability to generate resistance to different drugs. Both the ability of this cancer to spread and to generate resistance are mediated by the interactions between the tumor cells themselves and the rest of the elements present, both cellular and components of the extracellular matrix that form the tumor microenvironment (TME) [20].

The evolution of lung cancer in terms of the generation of resistance is variable and is linked to intrinsic factors of each patient, so there is a demand to generate study systems based on personalized medicine that allow us, on the one hand, to carry out a reliable screening of possible new pharmacological agents useful for the treatment of lung cancer, and on the other, predicting the response of each patient to treatment [21,22,23]. Thus, more and more researchers in the field of biology and engineering are exploring different 3D culture systems, which are much more representative of TME than traditional 2D cultures [24,25,26,27]. The study and understanding of the TME is essential for a better understanding of the disease, but the problem is that the complexity of the TME itself in terms of its composition (cancer cells, stromal cells, endothelial cells, macrophages, different types of lymphocytes, collagen and elastic fibers, macromolecules, etc.) make its study extremely difficult [28]. Among the cellular components of the TME, the role of CAFs has gained great importance in recent years. These cells condition tumor growth, the acquisition of resistance and control tumor migration through EMT regulatory mechanisms, in addition to conditioning the stroma to allow the nesting of tumor cells in other organs [29,30,31]. Although many resources have been used to study CAFs in recent years, the involvement of CAFs in the organization of cells in lung tumors requires further investigation.

In relation to the nomenclature, there is no unanimity regarding the denomination of organoids and spheroids. We initially applied the term spheroids to all cell groups with which we have worked, in agreement with other authors [18]. However, we have observed that the spheroids that contain CAFs or NFs acquire self-organization capacity, which is a characteristic of organoids [32]. For this reason, we have called these groups of cells organoids, keeping the term spheroid for those groups formed by a single cell type and which do not seem to have this capacity for self-organization.

Thus, this work is based on two fundamental objectives. On the one hand, we intend to study the role of CAFs in the evolution of lung tumors. To do this, we generated organoids using a reference tumor cell line, A549 cells, and we limited the composition of the cell matrix to the most relevant molecule in lung tumors, type I collagen [20,33]. With this strategy, we aim to eliminate complex factors, and thus be able to identify the role of CAFs more reliably in tumor evolution. Furthermore, we have generated organoids from fibroblasts isolated from healthy tissue of the same patients from which we have isolated the CAFs, in order to identify exclusive actions of these cells. On the other hand, we have tried to standardize which elements are relevant, from the morphological point of view, to settle the basis of a pharmacological model that could be generated from cells isolated from tumors of patients undergoing surgery, in order to have an idea of their possible response to treatment. For this reason, we optimized the culture conditions to generate organoids very quickly and thus, in a few days, be able to have an estimate of the best treatment for the patient.

We have focused on the morphological changes that affect both individual cells and, above all, those that affect the generated organoids, understanding them as entities. We have studied these cell groups using confocal microscopy, which has allowed us to reconstruct the 3D complexities of the organoids, as well as electron microscopy, which has allowed us to characterize the ultrastructural morphological changes of the cells—both the cells that form the organoids, as well as the cells that migrate through the matrix to generate new organoids—separately from the original ones. Although similar studies have been described in other models, we have not found previous studies that evaluate the parameters described here using CAFs and FNs obtained from NSCLC tumors.

The inclusion of spheroids in the present study has been carried out to provide reference parameters and thus be able to interpret the results generated in the culture of organoids. Regarding stromal cells, it is noteworthy that both NFs and CAFs only eventually generated compact clumps of cells, since they have a natural tendency to invade the surrounding stroma [34]. Therefore, the parameters observed in relation to their growth have been carried out on exceptional groupings of these cells and, therefore, must be considered carefully. CAFs generated cell clusters more frequently than NFs. This could be because CAFs represent a highly heterogeneous population that includes not only tumor-modified fibroblasts, but also mesenchymal cells, and even cancer cells with fibroblastic phenotypes [35]. Perhaps the presence of these cells could facilitate the formation of organoids. A549 cells always generated compact cell spheroids, which is consistent considering their epithelial origin. These spheroids always maintained a core of very cohesive cells and migratory cells appeared around them, in which a transitional morphology to mesenchymal cells could be observed. It is noteworthy that this cell line maintains a certain stromal phenotype, as demonstrated by the expression of vimentin, for example, being also capable of synthesizing TGF beta, a well-known EMT inductor, which could explain this circumstance [36].

The incorporation of stromal cells had a devastating effect on the morphology of the A549 organoids. Furthermore, these organoids were larger, and they grew faster than the spheroids. They showed a 3D organization, acquiring an alveolar morphology. We were able to observe how groups of migratory cells were generated from the organoids, led by pankeratin-negative fibroblast cells. These cells could be A549 that have fully transitioned to mesenchymal cells, with the consequent decrease in keratin expression. However, it is more reasonable to assume that they are stromal cells, which is consistent with the role assigned to these cells in relation to the control of tumor cells expansion. The cells in the central portion of the migrating cords were formed almost exclusively by epithelial cells that maintained the characteristic intercellular junctions. These changes were much more pronounced in CAF-containing organoids, where more clusters of migratory cells appeared, forming longer and noticeably more branched cords. Once again, the presence of fibroblast cells at the leading edge of these branches was evident, which reinforces the idea of the role of CAFs in tumor dissemination [37]. We found a striking difference in the profile of cells that appeared isolated around the organoid. In the case of CAFs, we observed a greater number of these cells, finding both epithelial cells in different stages of EMT and fibroblastic cells, often very close to each other, but without establishing intercellular junctions. We also observed small organoids in the proximity of larger ones, which suggests that there was a dissemination of cells in the first place that later reorganized to form new organoids, which is reasonable considering the role of CAFs in metastasis reported by other authors [38]. When we increased the proportion of NFs or CAFs, we observed organoids with more branches and a greater number of migratory cells, reinforcing the organizing concept of CAFs in the TME.

Most cells forming the organoids were secretory cells. Those inside the organoids showed a characteristic epithelioid phenotype, with multiple junctional complexes, abundant RER, Golgi apparatus, glycogen, and mitochondria. In the organoids, the cohesion of the internal cells was lower, displaying small discontinuities that formed lumens flanked by multiple cell processes. At the periphery of the organoids, cells exhibited several processes rich in actin filaments, especially in organoids that contained abundant CAFs. This notable increase in the number of cellular processes in the periphery of the organoid should have its effects at the level of scaffold stiffness, so it would be interesting to perform biomechanical studies to verify this [39]. This indicates the existence of a gradation in the cellular phenotype from the center of the organoid (epithelioid) to the periphery (fibroblastic). This internal organization of the organoids could be controlled by either one of the two cell types used to generate the organoids, so further experiments would be needed to identify the cell type that governs their dynamics. Regarding the deposit of extracellular matrix, we observed it in both organoids studied, being more notable in those containing CAFs, which is in line with the evidence reported by other authors.

In summary, the model proposed here could be a good model to study the effect of different drugs in relation to the growth and dissemination of lung tumors, which could be relevant when individualizing the treatment of patients with NSCLC. However, it is necessary to carry out pharmacological studies to verify if this model can really be relevant from the point of view of translational medicine.

## 4. Materials and Methods

### 4.1. Experimental Design

Adenocarcinoma stromal cells were isolated from patients diagnosed with lung adenocarcinoma (cancer-associated fibroblasts, CAFs), as well as from peripheral healthy tissue (normal fibroblasts, NFs). Cells were characterized via flow cytometry and expanded in 2D culture using proliferation medium. Spheroids were constructed using the drag drop method with A549 cells, CAFs or NFs. Organoids were constructed by mixing A549 cells and CAFs or NFs, using 10^5^ to 6 × 10^5^ cells, with a proportion of 50–75% stromal cells, after which they were cultured for up to one week. Cell assembly was monitored via phase contrast microscopy, images of each spheroid/organoid were acquired every 24 h, and the area of each spheroid/organoid was measured. The concentration of 1.5 × 10^5^ cells/mL and 72 h of culture were chosen as the best conditions, so they were used in the rest of the experiments.

Thus, spheroids or organoids of 72 h of culture were transferred to 20 µL droplets of type I collagen and cultured for an additional week. Morphology and cytoskeletal organization were determined via F-actin fluorescent staining using phalloidin-rhodamine. Immunofluorescence studies of pankeratin and vimentin were performed to determine the cell origin. Fluorescence images were acquired via epifluorescence confocal microscopy. In this case, 3D reconstruction of the acquired stacks was carried out to determine the morphology of the organoids. Real-time RT-PCR was performed to determine the relative gene expression levels of *CDH-1*, *CDH-2*, and *VIM*. Transmission electron microscopy was used to study ultrastructural features of cultured cells.

### 4.2. Cell Lines and Cell Isolation

The human lung adenocarcinoma epithelial cell line A549 was purchased from the American Type Culture Collection (ATCC, Rockville, MD, USA). Cells were cultured in A549 cell proliferation medium, composed of RPMI 1640 with stable glutamine (EuroClone, Siziano PV, Italy) supplemented with 5% heat-inactivated fetal bovine serum (HI-FBS, Gibco, Scotland, UK), 10 mM HEPES, 1% penicillin–streptomycin (P/S), and 1% amphotericin B (Euroclone, Siziano PV, Italy) in a humidified atmosphere incubator at 37 °C and 5% CO_2_.

Stromal cells were isolated from lung adenocarcinoma tissues of 3 different diagnosed patients who underwent surgery, as previously reported [40]. The samples were collected at Hospital La Ribera, in Alzira (Valencia, Spain). This study was conducted in accordance with the ethical standards of the Declaration of Helsinki of the World Medical Association. All the procedures included in this study were approved by the hospital ethics committee, and informed consent was obtained from all included patients. To obtain CAFs and NFs, tumor and non-tumor tissues, respectively, were fractionated into small pieces of approximately 3 mm and digested with DH liberase (Sigma-Aldrich, Madrid, Spain) for 90 min under shaking at 37 °C. Samples were filtered through a 70 µm filter (Gibco, Madrid, Spain) washed in sterile PBS (Sigma-Aldrich, Madrid, Spain) and cultured in fibroblast culture medium, composed of high-glucose DMEM culture medium supplemented with 10% HI-FBS, 1% penicillin–streptomycin (P/S), and 1% amphotericin B, in 6-well culture plates (Euroclone, Siziano PV, Italy) in the incubator to confluence. Adherent cells were expanded and subcultured for a maximum of 9 passages.

### 4.3. Formation of Organoids/Spheroids

Organoids or spheroids were constructed using the hanging drop method. Briefly, cell suspensions (10^5^ to 6 × 10^5^ cells/mL) were seeded as 50 drops (25 µL/drop, pipetting only to the first top of the pipette, to avoid bubble formation) onto the inside of lids of Petri dishes (87.8 mm diameter). Then, the lids with the hanging droplets were used to cover the Petri dishes containing 8 mL of PBS to generate a humidified atmosphere inside the dish. The hanging drops were cultured at 37 °C and 5% CO_2_ for up to 1 week.

### 4.4. Organoid/Spheroid Culture in Type I Collagen Hydrogels

After 72 h of culture, the organoid/spheroid were collected using a sterile Pasteur pipette and transferred to a 50 mL conical flask containing DMEM F12 supplemented culture medium. The organoids/spheroids were incubated for 10 min at room temperature (RT) and the supernatant was removed. The pellet with the organoids/spheroids was resuspended in 450 µL of an ice-chilled solution containing 4 mg/mL rat type I collagen (Advanced Biomatrix, Carlsbad, CA, USA) and mixed with 50 µL of neutralizing solution (Advanced Biomatrix, Carlsbad, CA, USA). Then, 20 µL drops were transferred to a well of a 24 well untreated culture plate (Corning, NY, USA), and the collagen droplets containing spheroids were incubated for about 20 min at 37 °C in an incubator to allow jellification of the collagen, after which culture medium was added to cover up the droplets. The culture medium was changed every 2–3 days and the organoids/spheroids were photographed daily to study cell morphology using a Leica DM IL LED phase contrast microscope (Leica Microsystems, Wetzlar, Germany) for one week.

### 4.5. F-Actin Fluorescence Staining and Vimentin and Pankeratin Proteins Expression and Distribution in Organoids/Spheroids

F-actin was evaluated using rhodamine-conjugated phalloidin (Molecular Probes, Thermo Fisher Scientific, Madrid, Spain) as previously described [41]. Protein expression of vimentin and pankeratin was determined using specific antibodies. Organoids/spheroids were cultured for one week as described above in the type I collagen gels, and then the droplets were transferred to 8-well Millicell slides (Merck, Darmstadt, Germany) or to non-adherent culture dishes [Corning, NY, USA] for visualization using epifluorescence or confocal microscopy, respectively.

Cells were washed with PBS pH 7.4 for 5 min twice and fixed with 4% paraformaldehyde solution in PBS for 20 min at RT. After washing twice with PBS, cells were permeabilized with 0.1% Triton X 100 in PBS for 3–5 min and, after two washes with PBS, preincubated with blocking solution (1% BSA in PBS) for 20–30 min to reduce non-specific background.

For phalloidin staining, sample were incubated for 1 h with phalloidin (5 µL phalloidin stock solution in DMSO diluted in 200 µL PBS).

For vimentin or cytokeratin immunodetection, samples were incubated with the appropriate monoclonal antibodies diluted in antibody diluent solution. The antibodies used were the primary monoclonal vimentin antibody (2D1; 1:500 dilution; Novus Biologicals, Englewood, CO, USA) and Pan-Alexa fluor cytokeratin (1:100 dilution; Invitrogen, Waltham, MA, USA). Samples were incubated with the antibody overnight at 4 °C and then washed 3 times with PBS. For vimentin detection, samples were then incubated for 1 h at RT with the anti-mouse FITC-conjugated secondary antibody (Sigma-Aldrich, Madrid, Spain) diluted 1:200. Visualization of cytokeratin was performed via direct immunofluorescence.

Finally, samples were washed several times with PBS, the nuclei were stained with DAPI and the samples were analyzed with a Leica DM2500 fluorescence microscope (Madrid, Spain). Cell morphology was studied using the distribution of F-actin microfilaments.

### 4.6. Transmission Electron Microscopy (TEM)

A transmission electron microscopy (TEM) study was performed to examine the ultrastructure of the spheroids/organoids. After 72 h organoid/spheroid culture, samples were washed with PBS and fixed in ice-cold 2.5% glutaraldehyde. Subsequently, the organoids were post-fixed in 1% osmium tetroxide overnight at 4 °C, washed and dehydrated through a standard ethanol series. The samples were then embedded in epoxy resin and cut into 60 nm semithin sections. Finally, ultrathin sections were double stained with 3% uranyl acetate and lead citrate. Ultrastructure images were obtained with a transmission electron microscope (Hitachi 800, Tokyo, Japan).

### 4.7. Determination of Relative Gene Expression Levels of CDH1, CDH2 and VIM

Total RNA was extracted from the samples after 72 h of culture in the type I collagen gel using TRIzol reagent (Thermo Fisher Scientific Inc., Waltham, MA, USA), according to the manufacturer’s instructions. RNA concentration was determined via spectrophotometry using a Nanodrop 2000 spectrophotometer (Fischer Scientific, Madrid, Spain). RNA integrity was assessed via capillary electrophoresis using a Bioanalyzer (Agilent Technologies, Santa Clara, CA, USA). Only extracts with a 260/280 nm ratio > 1.8 and with RIN of ~10 were used for the determination of gene expression levels.

Random hexamers were used to synthesize complementary DNA (cDNA) using TaqMan RT reagents (Applied Biosystems, Foster City, CA, USA), following the manufacturer’s instructions. Gene expression levels were assayed via reverse transcriptase polymerase chain reaction (RT-PCR) using Assays on Demand (Applied Biosystems, Madrid, Spain). The reactions were carried out in a 7900HT Real-Time Thermocycler (Applied Biosystems, Madrid, Spain) [36]. The comparative ΔΔCt method with glyceraldehyde 3 phosphate dehydrogenase (*GAPDH*) was used as an endogenous control to calculate relative levels of gene expression.

### 4.8. Morphometric Study

The 3D structure of the generated spheroids/organoids was reconstructed from serial images obtained via confocal microscopy (LEICA TCS-SP8, Leica Corp., Madrid, Spain) according to the following parameters: using a 10× objective lens and determining the number of steps of the stacks as half of the optimized number of steps given by the system. Videos were created from image stacks acquired with Imaris Microscopy Image Analysis Software, Version 9.0 (Oxford Instruments, Abingdon, UK). The dimensions of the spheroid in the Z plane (height) were estimated by measuring with the tool “draw scalebar” on the orthogonal view ZY, the distance between the lowest point and the highest point of the spheroid/organoid.

### 4.9. Data Presentation and Analysis

Cells from 3 different patients were used for NFs and CAFs isolation, and all determinations were carried out in triplicate. For microscopy experiments, representative images of 5 fields of each stain are presented. Data are presented as the mean ± SD. Statistical analysis was carried out using analysis of variance (ANOVA) followed by Tukey’s multiple-comparison test (GraphPad Software Inc., San Diego, CA, USA). Significance was accepted at *p* < 0.05.

## Data Availability

Not applicable.

## Supplementary Materials

The supporting information can be downloaded at: https://www.mdpi.com/article/10.3390/ijms241210131/s1.
